# Adrenal Volume Quantitative Visualization Tool by Multiple Parameters and an nnU-Net Deep Learning Automatic Segmentation Model

**DOI:** 10.1007/s10278-024-01158-y

**Published:** 2024-07-02

**Authors:** Yi Li, Yingnan Zhao, Ping Yang, Caihong Li, Liu Liu, Xiaofang Zhao, Huali Tang, Yun Mao

**Affiliations:** 1 Department of Radiology, The First Affiliated Hospital of Chongqing Medical University, Chongqing, 400016 China; 2 Guangzhou University, Guangzhou, China

**Keywords:** Adrenal gland, Volume quantitative, Convolutional neural network, nnU-Net, Image segmentation

## Abstract

Abnormalities in adrenal gland size may be associated with various diseases. Monitoring the volume of adrenal gland can provide a quantitative imaging indicator for such conditions as adrenal hyperplasia, adrenal adenoma, and adrenal cortical adenocarcinoma. However, current adrenal gland segmentation models have notable limitations in sample selection and imaging parameters, particularly the need for more training on low-dose imaging parameters, which limits the generalization ability of the models, restricting their widespread application in routine clinical practice. We developed a fully automated adrenal gland volume quantification and visualization tool based on the no new U-Net (nnU-Net) for the automatic segmentation of deep learning models to address these issues. We established this tool by using a large dataset with multiple parameters, machine types, radiation doses, slice thicknesses, scanning modes, phases, and adrenal gland morphologies to achieve high accuracy and broad adaptability. The tool can meet clinical needs such as screening, monitoring, and preoperative visualization assistance for adrenal gland diseases. Experimental results demonstrate that our model achieves an overall dice coefficient of 0.88 on all images and 0.87 on low-dose CT scans. Compared to other deep learning models and nnU-Net model tools, our model exhibits higher accuracy and broader adaptability in adrenal gland segmentation.

## Introduction

### Clinical Background

Adrenal size abnormalities can indicate a variety of diseases, such as adrenal hyperplasia, adrenal adenoma, and adrenal cortical adenocarcinoma [1–5]. Timely diagnosis and treatment contribute to improving the cardiovascular and renal status of patients with these diseases, correcting metabolic and electrolyte imbalances, and reducing the risk of associated complications [6–8]. Currently, the radiological diagnosis of these diseases relies on subjective evaluation using the cross-sectional mode and linear adrenal gland measurements [3, 4, 9, 10]. However, the adrenal gland poses a challenge due to its small size and intricate boundaries, exhibiting considerable variability in shape and position. Manual diagnosis is prone to omissions, significant variations in measurement data, and poor interobserver consistency. For instance, the interobserver agreement of limb linear measurements ranged from 0.1869 to 0.7638. Additionally, the diagnostic criteria for hyperplasia based on 2D linear measurements differ significantly, rendering the sensitivity and specificity unreliable. In fact, at a specificity of 100%, the sensitivity is merely 47% [11]. Consequently, the application of 2D linear adrenal measurements in clinical practice is constrained. Moreover, relying solely on linear evaluation represents a one-dimensional approach that fails to capture the intricate three-dimensional morphology of the organ. Consequently, adrenal volume emerges as the most comprehensive indicator of adrenal size [2, 9, 12, 13]. Adrenal volume has also been shown to be closely related to adrenal-related endocrine diseases, hypothalamic pituitary adrenal axis HPA-related diseases, obesity, impaired glucose metabolism, polycystic, and septic shock. It is one of the indicators reflecting these diseases [11, 14–18]. Monitoring adrenal volume can provide a radiological quantitative indicator for these diseases. In addition, quantitative screening of patients with enlarged adrenal volume may improve the early diagnosis of subclinical patients with adrenal cortical hyperplasia, thereby reducing the risk of related metabolic and cardiovascular diseases [19–22]. Adrenal volume can also provide supplementary information for calculating the severity of different types of adrenal hyperplasia [13]. Adrenal volume measurement is also a critical determinant of unilateral adrenalectomy. The surgical effect of unilateral adrenalectomy based on adrenal CT measurement values is similar to that found on adrenal venous blood collection [23]. In summary, adrenal volume has crucial clinical significance, and it is vital to develop an automatic adrenal volume quantification visualization tool. However, automatically segmenting the adrenal gland is challenging compared to other large abdominal organs.

## Related Work

Previously, estimating adrenal volume used manual contouring methods, which are not only labor-intensive processes but their repeatability and consistency could be better. With the development of deep learning algorithms, adrenal segmentation and volume assessment are also being explored to be fully automated, which may be more efficient and accurate than manual linear evaluation.

Traditionally, Bhole et al. proposed a conditional random field to learn edge distribution parameters in 2013 [25]. In 2015, Chai et al. introduced multi-scale sparse representations as a method to achieve precise adrenal tumor contouring [24]. In 2018, Koyuncu et al. explored region growing techniques for adrenal segmentation [26]. Additionally, Zhang and Li utilized level set methods in 2019 for the same purpose [27]. However, traditional segmentation methods primarily rely on handcrafted features, which inherently lack the ability to generate high-level representations. As a result, these methods often yield suboptimal segmentation accuracy. Robinson et al. utilized a U-Net based segmentation model for adrenal segmentation in their study. They conducted their research using a development dataset consisting of 274 CT examinations from 251 patients. Additionally, a secondary test set containing 991 CT examinations from 991 patients was employed. The model achieved a median dice similarity coefficient (DSC) of 0.80 for normal adrenal glands on the development test set [28]. In a similar vein, Kim et al. also employed a U-Net based segmentation model for adrenal segmentation. The model yielded DSC of 0.699 for the ordinary group and 0.706 for the abnormal group [29]. However, the lack of targeted design in these approaches has resulted in suboptimal performance. Moreover, it is worth noting that the model’s accuracy was constrained by its reliance on enhanced venous phase CT images and the absence of external validation. Furthermore, Luo et al. proposed a two-level cascade deep neural network for adrenal segmentation using a dataset of 348 CT images. Their study found no significant difference in average volume between the reference standard and the predicted mask (*P* = 0.06). However, it is important to note that they specifically used premedication CT images of patients with primary aldosteronism, and further investigation is required to determine the generalizability of this model to the broader population [30]. In 2024, Li et al. introduced a novel multi-level context-aware network for adrenal gland segmentation in CT images. Their approach achieved DSC of 0.7134 and 0.7529 on two datasets [31]. However, it is noteworthy that the two-stage network employed in their study is slower compared to a single-stage network, particularly when dealing with large-scale image data. Additionally, the two-stage network typically involves training multiple models simultaneously, which adds complexity and scalability challenges to the process. Utilizing solely a singular parameter dataset and the inherent constraints of model performance hinder their evolution into a universally precise automatic tool for daily clinical application.

### Rationale

U-Net has become the benchmark in medical image segmentation [32]. The no new U-Net (nnU-Net) derived from U-Net is an adaptive convolutional neural network framework [33]. It has improved the architecture of automatic medical image segmentation and achieved good results in various international competitions [34–39].The structure of U-Net consists of an encoder and a decoder, following a U-shaped design. The encoder progressively down samples to extract features, while the decoder progressively up samples to restore spatial resolution, enabling precise segmentation. nnU-Net cross-layer connections integrate features across scales for prediction; allowing U-Net to accurately identify minute anatomical structures in medical images is a framework that automatically optimizes U-Net hyperparameters by adjusting network configurations and training strategies based on the characteristics of the dataset, enhancing U-Net’s efficiency and accuracy across various segmentation tasks. It incorporates various methods, including cross-validation and data augmentation, to ensure comprehensive training and evaluation, making U-Net even more precise in medical image segmentation.

Our research aims to create a comprehensive, accurate, and fully automated tool for visualizing and quantifying adrenal volume. This tool is designed to be universally applicable, leveraging a large dataset, multiple parameters, various machine types, diverse radiographic doses, varying layer thicknesses, numerous scanning methods, different phase images, and a nnU-Net deep learning automatic segmentation model. Our goal is to fulfil daily clinical practice and scientific research requirements.

## Materials and Methods

### Patient Data

This retrospective study, which complied with the principles outlined in the Declaration of Helsinki, received approval from the Ethics Committee of the first affiliated hospital of chongqing medical university under approval number K2023-417. As it involved a review of past data, it was deemed exempt from informed consent requirements by the institutional review board. The study included 885 CT images from patients who underwent medical examinations at first affiliated hospital of chongqing medical university between January 2018 and December 2022, as well as 280 CT images from patients who underwent medical tests at the first branch and Jinshan hospital of chongqing medical university.

**Fig. 1:**
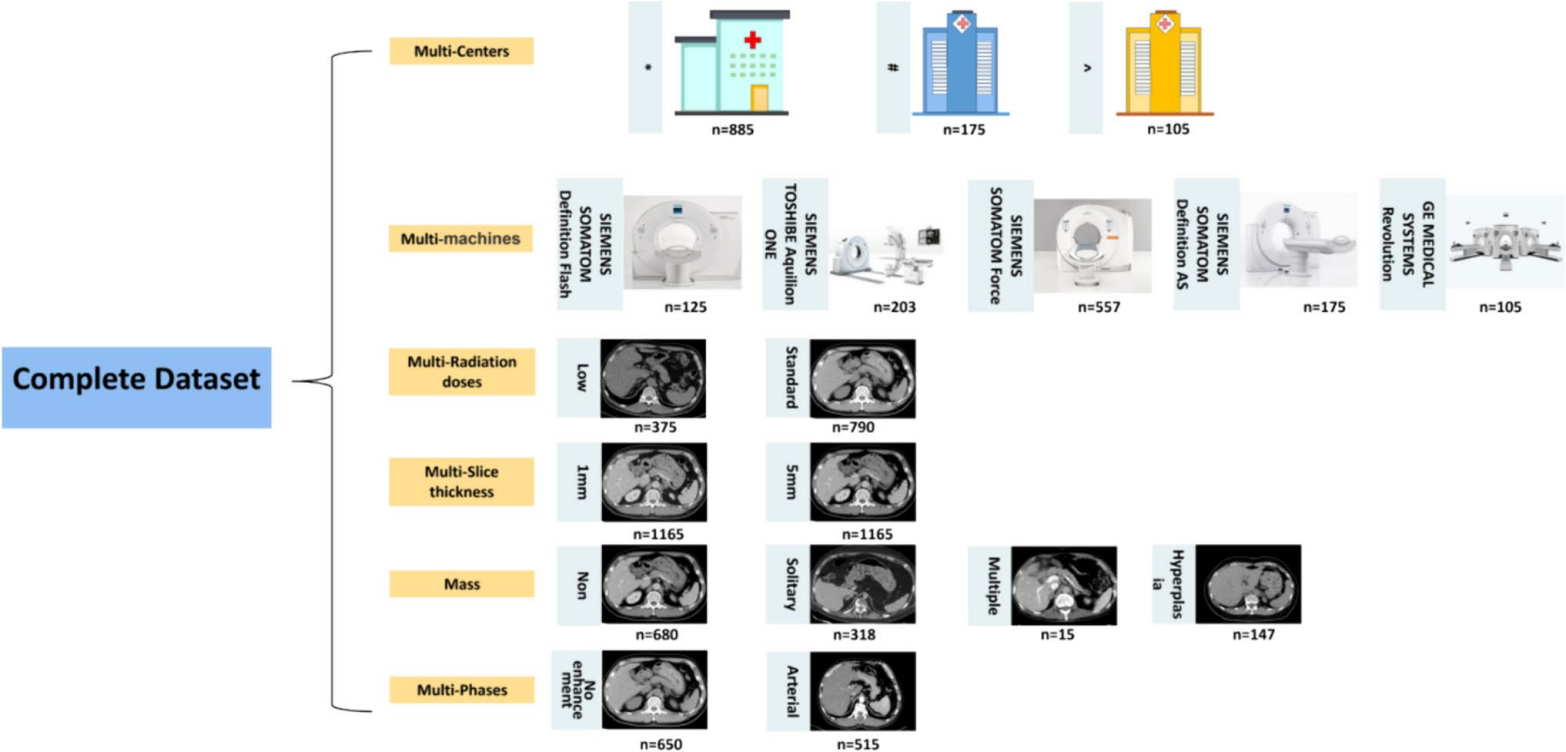
Detailed data composition

The CT images of normal adrenal glands included in the study met the following criteria: (1) patients who underwent low-dose chest plain CT, routine-dose abdominal contrast-enhanced CT, or abdominal plain CT and (2) formal radiology reports that indicated “no abnormalities in the adrenal glands” or “normal adrenal glands.” A total of 680 patients were included with CT images. The CT images of patients with adrenal hyperplasia included in the study met the following criteria: (1) patients who underwent low-dose plain chest CT, routine-dose abdominal contrast-enhanced CT, or plain abdominal CT and (2) formal radiology or pathology reports that indicated “adrenal hyperplasia” or “enlarged adrenal glands.” A total of 150 patients were included with CT images. The CT images of patients with adrenal nodules included in the study met the following criteria: (1) patients who underwent low-dose chest plain CT, routine-dose abdominal contrast-enhanced CT, or abdominal plain CT and (2) formal radiology or pathology reports that indicated “adrenal nodules”, “adrenal tumours,” “adrenal adenomas,” “adrenal carcinomas,” “adrenal cysts,” or “adrenal medullary tumours.” A total of 335 patients were included with CT images. The detailed data composition is shown in Fig. 1.

**Fig. 2:**
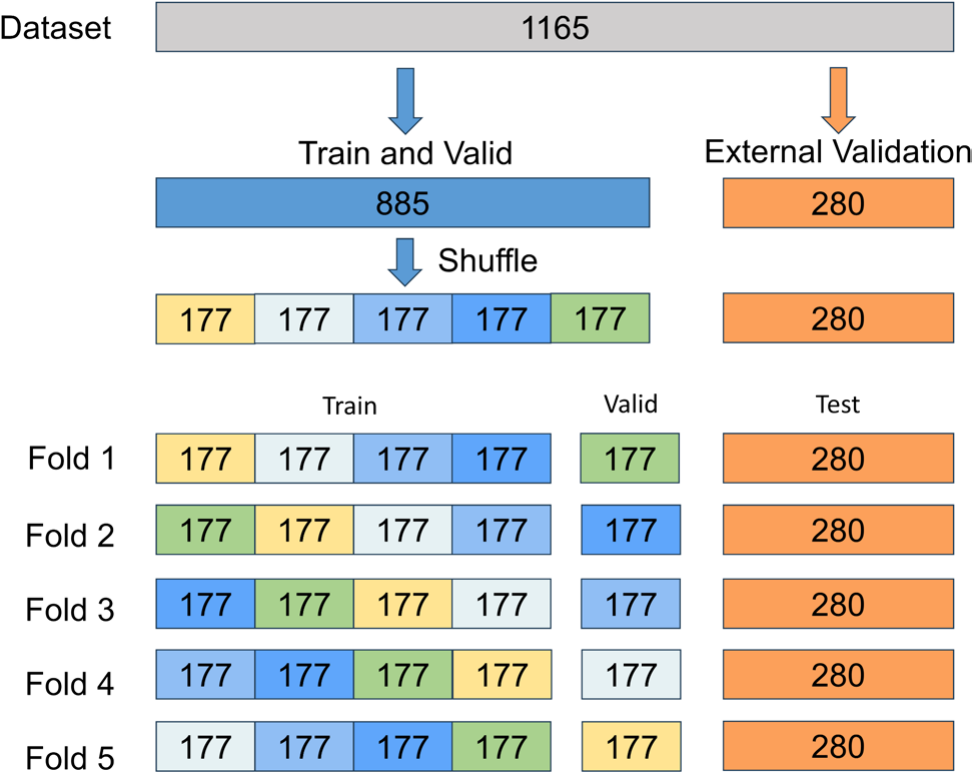
Dataset partitioning diagram

The 885 CT images from patients who visited and underwent medical examinations at the* were randomly allocated by a computer in an 8:2 ratio to form the validation set of 708 cases and the internal validation set of 177 cases. The external validation set included 280 CT images from patients who visited and underwent medical examinations at # and ^. The detailed dataset partitioning is shown in Fig. 2.

### CT Equipment and Scanning Parameters (Table 1)

**Table 1:** Computed tomography equipments and scanning parameters

CT equipment	Scanning site and mode	Tube potential (KVp)	Scanner pitch	Kernel	Slice thickness (mm)	Scanning matrix
SIEMENS HEALTHCARE SOMATOM Definition Flash	Abdominal enhanced scan	120	1.0	Br30f	1\5	512 × 512
SIEMENS HEALTHCARE TOSHIBE Aquilion ONE	Abdominal plain scan	120	1.0	Br30f	1\5	512 × 512
	Abdominal enhanced scan	120	1.0	Br30f	1\5	512 × 512
SIEMENS HEALTHCARE SOMATOM Force	Chest low-dose scan	100	1.0	Br40/BI57	1\5	512 × 512
	Abdominal plain scan	120	1.0	Br40	1\5	512 × 512
	Abdominal enhanced scan	120	1.0	Br40	1\5	512 × 512
SIEMENS HEALTHCARE SOMATOM Definition AS	Chest low-dose scan	100	1.0	Br35/BI40	1\5	512 × 512
	Abdominal plain scan	120	1.0	Br20f	1\5	512 × 512
	Abdominal enhanced scan	120	1.0	Br30f	1\5	512 × 512
General Electric MEDICAL SYSTEMS Revolution	Abdominal plain scan	120	1.25	Br20f	1.25\5	512 × 512
	Abdominal enhanced scan	120	1.25	Br30f	1.25\5	512 × 512

The chest low-dose scan dose CTDIvol<1.5 mGy, and the remaining scan doses comply with the American College of Radiology guidelines for routine scans

We used arterial phase images, which were acquired by injecting iodinated contrast agent 350 mGI/mL into the peripheral veins of the upper extremities using an automatic power injector at a total dose of 1.5 mL/kg, obtained within 30 s. A SIEMENS HEALTHCARE SOMATOM Force device was exclusively used for conducting low-dose chest CT examinations in the training and internal validation sets. In contrast, three devices were utilized for abdominal contrast-enhanced CT examinations: the SIEMENS HEALTHCARE SOMATOM Definition Flash, TOSHIBA Aquilion ONE, and SIEMENS HEALTHCARE SOMATOM Force, each contributing to at least one-quarter of the total cases. Two devices were utilized for abdominal plain CT scans: the TOSHIBA Aquilion ONE and SIEMENS HEALTHCARE SOMATOM Force, each accounting for no less than one-third of the total cases. The external validation set incorporated the SIEMENS HEALTHCARE SOMATOM Definition AS+ and the GE MEDICAL SYSTEMS Revolution (Table 2).

**Table 2:** Specific grouping data for the training set and validation set

Type	Training set and internal verification set (*n* = 885)	External verification set (*n* = 280)
Normal chest low-dose plain scan 5 mm	200	35
Normal chest low-dose plain scan 1 mm	200	35
Abnormal chest low-dose plain scan 5 mm	120	20
Abnormal chest low-dose plain scan 1 mm	120	20
Normal abdominal normal-dose plain scan 5 mm	100	55
Normal abdominal normal-dose plain scan 1 mm	100	55
Abnormal abdominal normal-dose plain scan 5 mm	90	30
Abnormal abdominal normal-dose plain scan 1 mm	90	30
Normal abdominal normal-dose enhanced scan 5 mm	200	90
Normal abdominal normal-dose enhanced scan 1 mm	200	90
Abnormal abdominal normal-dose enhanced scan 5 mm	170	50
Abnormal abdominal normal-dose enhanced scan 1 mm	170	50

### Building the Dataset

A junior radiologist manually segmented the adrenal gland parenchyma on transverse images in all CT scans using the commercial software ITK-SNAP(3.8). The adrenal gland margins were drawn close to the gland surface to exclude adjacent fat tissue, adrenal vein, inferior vena cava, spleen, and pancreatic tail. All surgeries were performed within the standard adrenal window width of 300, level 40. A senior radiologist with 22 years of abdominal imaging experience reviewed the regions of interest.

### Image Preprocessing

The window width and level for the preprocessing step were set to 300 and 40, respectively. Given that the slice count for 5 mm data ranged from 33 to 97 with an average of 59 and the 1 mm data ranged from 65 to 725 with an average of 338, there were considerable differences in the *z*-axis number of slices and voxel spacing. We used the SimpleITK library to check the data’s spacing, origin, and direction, ensuring that every column of images and annotations was located within a perfectly matched voxel space. Afterwards, we reset the window width and level according to the clinical guidelines for the adrenal window, helping to speed up training and increase image contrast to enhance the training’s directionality. To achieve fully automated volume assessment for data with different slice thicknesses, we used a threshold of 3 mm to automatically categorize the slice thickness of the data to be processed, determining which model would be used to infer the segmentation results. We cropped the data in the all-zero area to reduce the data size further and minimize the interference of redundant information on training. After data cropping, we calculated the median voxel spacing for the entire dataset based on the total sample and resampled the samples to the same voxel space as the median. We then updated the voxel spacing, calculated the new sample shapes, and employed third-order spline interpolation for the images to the unique shape. Corresponding masks used nearest-neighbor interpolation. Finally, we computed the intensity value distribution of the entire dataset foreground and compressed the intensity values of all samples to the overall distribution’s [0.5, 99.5] percentile avoiding the impact of individual outliers on the comprehensive dataset, and we used the *Z* score normalization method to normalize the intensity values of all samples to a range of 0–1, making the model training process more stable.

### Image Segmentation and Data Training

**Fig. 3:**
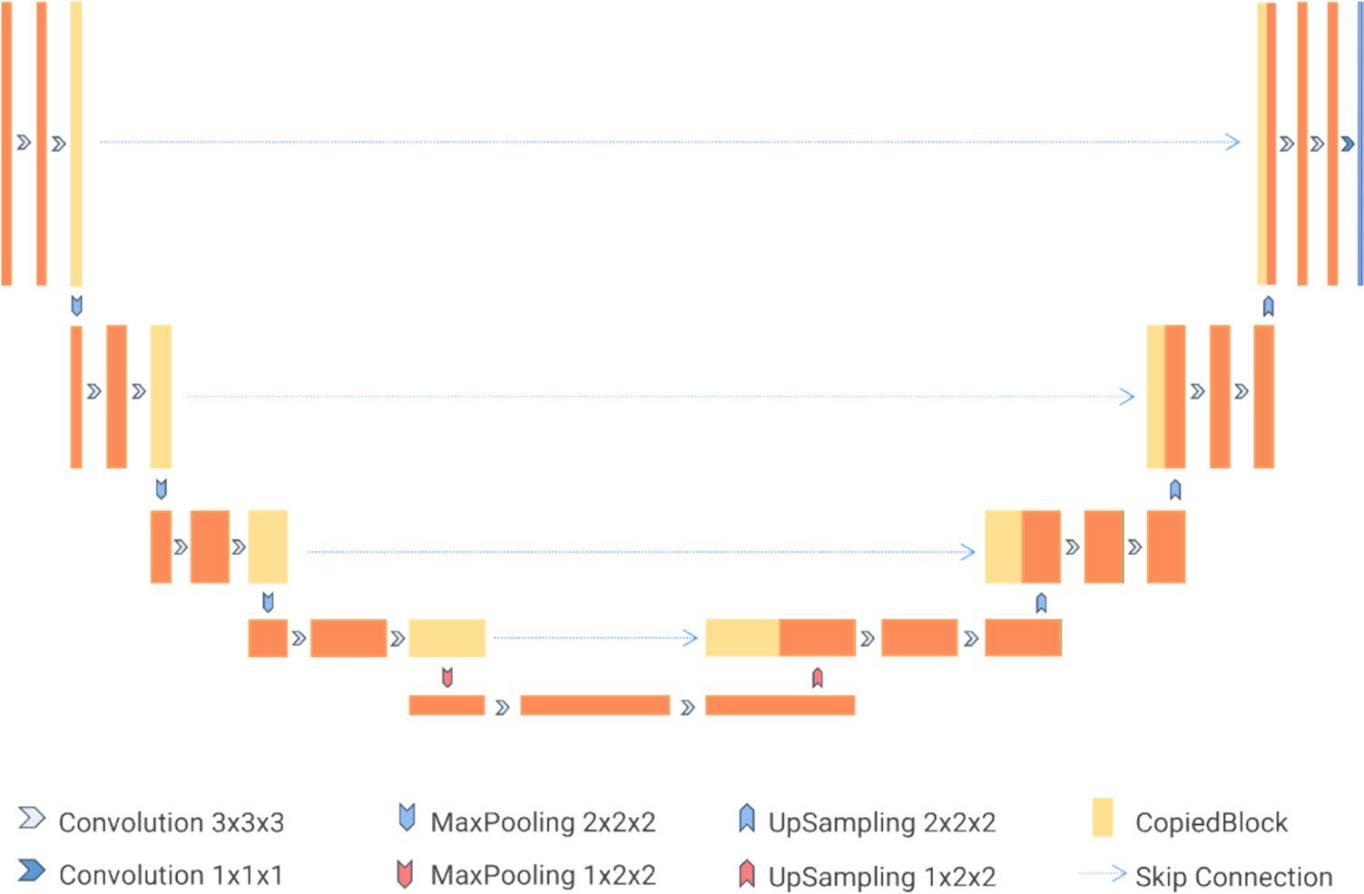
The network’s overall structure comprises encoder layers, each consisting of two consecutive convolutional layers followed by a maxpooling layer. Each convolutional layer incorporates batch normalization (BatchNorm) and leaky rectified linear unit (LeakyReLU) operations. The input to the network consists of patches sampled from the original image. The output is a segmentation mask encoded in a one-hot format. Depending on the specific patch, slight architectural differences exist between thick and thin-layer models

We used nnU-Net, which inherits the U-shaped U-Net for the overall network architecture. The structure can be divided into encoding and decoding paths. In the encoding path, each level uses two consecutive 3 × 3 × 3 convolutional layers and a 2 × 2 × 2 max pooling layer to gather semantic image features of the image. The decoding path then uses transposed convolutional layers of the same size as the pooling layers to restore the image to its original resolution. Each encoding path level output is skip-connected to the decoding path, aggregating the image’s high- and low-resolution features. The number of channels on the top layer is 32, and each layer doubles the number of input channels, but it does not exceed 320. To guarantee feature map accuracy on the lower layer, the three dimensions of this layer will not be smaller than 4. This is achieved by establishing the downsampling stride of one axis as 1. In the encoding path, each layer (except the last one) consists of two convolutional layers and one max pooling layer. The convolutional layers extract latent semantic features from the input image, while the pooling layers reduce the spatial dimensions of the feature maps, thereby decreasing the computational load and number of parameters for each layer and improving the model's efficiency and performance. The encoding path has five levels, each using a specific number of feature map channels: (32, 64, 128, 256, 320). To ensure that the feature maps are no smaller than 4 (to retain effective spatial information), the pooling and transposed convolution kernel sizes of the final network layer on the low-resolution axis are set to 1. Corresponding to the encoding path is the decoding path, in which each layer consists of an upsampling layer implemented with a transposed convolution and two successive convolutional layers. Transposed convolution increases the feature map size from a smaller spatial resolution to a larger one, which is useful for recovering image details and generating high-resolution outputs. Furthermore, skip connections are used between each corresponding encoder and decoder layer to merge low- and high-level features and pass gradients, which helps maintain stability during deep training. In the decoder’s final layer, a 1 × 1 × 1 convolution layer is used to map the number of feature map channels to the output channels. The complete network structure is shown in Fig. 3, with input as a 3D array composed of adrenal CT images and output as the adrenal segmentation mask. The complete network structure is illustrated in Fig. 3, and its input is the 3D array consisting of adrenal CT images.

We divided the 885 cases into five equal, non-overlapping folds for training. We used four folds each time for training and the remaining one for validation. Our model training was based on patches. To balance memory consumption and training time, the final chosen patch sizes for 5 mm and 1 mm were (28, 256, 256) and (40, 240, 240), respectively. The batch size was set to 2, and we used SGD [40] with a momentum of 0.99 and an initial learning rate of 0.01 as the optimizer. The learning rate used the “poly” policy [41] for dynamic adjustment with each epoch. We performed 250 iterations on randomly selected patches in each epoch. Before each iteration, there was a 50% chance to trigger five types of data augmentation [42]: random rotation, random scaling, random elastic transformation, gamma correction enhancement, and mirroring allowing the model to learn more abstract features to improve the model’s generalization ability further. We used a combination of Dice and cross-entropy loss to address the extreme class imbalance in medical image data for loss function selection. In addition, we trained the network using deep supervision [43], calculating weighted loss for feature maps of different scales at each level and setting their weights to decrease at a rate of 2. We trained each model for 1,000 epochs on a 3,090 model with 24 G of video memory, a typical epoch that took approximately 70 s.

After completing the fivefold model training, the external validation data used patches based on preprocessing and performed sliding window predictions with a 0.5 overlap. Since patches closer to the center received more iterations during training, our inference process assigned higher confidence to patches closer to the center.

### Volume Estimation

To calculate the physical volume of voxels in real space, we used the product of the in-plane and out-of-plane distances for each voxel contained in the CT data metadata header. Our volume estimation was based on this calculation. First, we used the SimpleITK library to obtain the voxel spacing information of the CT data. Then, we utilized the NumPy library to calculate the number of nonzero voxels in the data. Multiplying these two values gives us the physical volume of the data instance.

### Contrast Model

We conducted a within-model and cross-model comparison of the models. For the within-model comparison, we selected TotalSegmentator. To comprehensively evaluate the performance of different architectures, the cross-model comparison included the V-Net model based on convolutional neural networks and the Swin-UNETR model based on the Transformer architecture. To ensure fairness, we applied the same preprocessing strategies and training configurations, including patch size, data augmentation strategies, and loss functions, to the other two models as we used with our model. TotalSegmentator is a tool trained with the nnU-Net model to segment over 117 categories in CT images. It is trained on various CT images from different scanners, institutions, and protocols, making it suitable for segmenting organs in most CT images. The tool was trained on 1204 CT images with whole-body annotations, using 1.5-mm and 3.0-mm slice thicknesses, for 4000 epochs with nnU-Net. It achieved an average Dice score of 0.943 on an external validation set and has been widely applied in preliminary medical image segmentation tasks. V-Net is a Convolutional Neural Network architecture specifically designed for three-dimensional medical image segmentation tasks. Proposed by Fausto Milletari and colleagues in 2016, it addresses the challenge of automatically segmenting organs and lesions in 3D medical images, such as CT or MRI scans [44–46]. Swin-UNETR is a novel neural network model based on the Transformer architecture, proposed by Liu et al. in 2021 [47–49]. It is designed for computer vision segmentation tasks and introduces a mechanism called “shifted window” to efficiently process image data. The core innovation of this model lies in its hierarchical Transformer, which limits the self-attention mechanism to operate within local windows, reducing computational complexity and enhancing processing efficiency.

### Statistical Analysis

Statistical analysis and data processing were performed using Python 3.9 and SPSS 25.0 (IBM, Armonk, NY, USA). For quantitative data that follows a normal distribution, the mean ± standard deviation is used. For quantitative data not following a normal distribution, the median and interquartile ranges (IQRs) are employed. Qualitative data is reported as counts or percentages. Internal validation results are presented as the mean ± standard deviation (SD) on the fivefold cross-validation sets. In contrast, external validation results are reported as the mean ± SD of the entire external validation dataset. Chi-square or Fisher’s exact test is applied to evaluate qualitative data. The *t*-test, analysis of variance (ANOVA), or Kruskal-Wallis test is used to assess continuous variables. We assessed segmentation performance using various metrics: DSC, relative volume error (RVE), and the 95th percentile of the Hausdorff distance (HD95). These metrics quantified similarity and accuracy compared to ground truth data. DSC measured similarity between predicted and reference masks, indicating segmentation model performance on a scale from 0 to 1. Higher values represent better accuracy. HD95 measured boundary segmentation accuracy, while RVE evaluated volume accuracy in segmentation results.

The calculation formula for the RVE is as follows:

2-1 $$\text{RVE}=\frac{abs\left(\left|S\right|- \left|GT\right|\right)}{|GT|}$$

In the equation above, *S* represents the segmentation result, while GT represents the ground truth.

The RVE represents the relative error between the volume in the segmentation result and the volume of the actual object, expressed as a percentage.

2-2 $$HD\left(P, G\right)=max\left(h\left(P, G\right), h\left(G, P\right)\right)$$

2-3 $$h(P, G) =\underset{pi\in P}{max} \;\underset{gi\in G}{min}\| pi - gi \|$$

In the given equation, *P* represents the set of pixels enclosed by the segmentation contours, and *G* represents the set of pixels enclosed by the ground truth contours. Importantly, HD95, similar to HD, measures the Hausdorff distance but utilizes the 95th percentile instead of the maximum value as defined in the equation.

The intraclass correlation coefficient (ICC) was used to evaluate the volume consistency among different methods. ICC values were interpreted as follows: ICC < 0.5 indicates poor reliability, 0.5 ≤ ICC < 0.75 indicates moderate reliability, 0.75 ≤ ICC ≤ 0.9 indicates good reliability, and ICC > 0.9 indicates excellent reliability [50]. The calculation methods of ICC vary depending on different research designs and application contexts. The most common formulas are based on mixed-effects models or random-effects models. Two typical calculation methods are as follows:

1. Single-measure form ($\text{ICC}(\text{1,1})$).
  Used to assess the reliability of a single instance of measurement by a single rater or evaluator on the same set of samples, the calculation methods are as follows:
  2-4 $$\text{ICC}(\text{1,1})=\frac{M\;\cdot\; {S}_{B}-M\;\cdot\; {S}_{W}}{M\;\cdot\; {S}_{B}+(k-1)\;\cdot\; M\;\cdot\; {S}_{W}}$$
2. Average-measure form ($\text{ICC}(1,k)$).
  Used to assess the consistency of the average across multiple repeated measurements, the calculation methods are as follows:
  2-5 $$ICC(1,k)=\frac{M\;\cdot\; {S}_{B}-M\;\cdot\; {S}_{W}}{M\;\cdot\; {S}_{B}+(1/k)\;\cdot\; M\;\cdot\; {S}_{W}}$$

Among these, $M\;\cdot\; {S}_{B}$ is the between-group mean square, reflecting differences between different groups. $M\;\cdot\; {S}_{W}$ is the within-group mean square, indicating differences between measurements within a group. $k$ is the number of measurements per group.

## Results and Experiment

### Clinical Baseline Information

A total of 1165 patients were included, with 885 cases in the training and internal validation set, and 280 cases in the external validation set. The average age of the patients was 54 years old, and the distribution of adrenal morphology categories was even across all datasets (all *P* > 0.05). The detailed is shown in Tables 3 and 4.

**Table 3:** The comparison of clinical baseline information between internal and external test sets

	Total (*n* = 1165)	Internal (*n* = 885)	External (*n* = 280)	Statistic	*P*
Age, mean ± SD	53.85 ± 13.23	54.07 ± 13.01	53.17 ± 13.93	*t* = 0.99	0.324
Sex, *n* (%)				*χ*^2^ = 2.44	0.118
Female	427 (36.78)	335 (38.02)	92 (32.86)		
Male	734 (63.22)	546 (61.98)	188 (67.14)		
Status, *n* (%)				*χ*^2^ = 5.31	0.070
Hyperplasia	150 (12.88)	119 (13.45)	31 (11.07)		
Nodule	335 (28.76)	266 (30.06)	69 (24.64)		
Normal	680 (58.37)	500 (56.50)	180 (64.29)		
Nodule characteristics, *n* (%)				*χ*^2^ = 0.00	0.956
Malignant	7 (2.09)	5 (1.88)	2 (2.90)		
Benign	328 (97.91)	261 (98.12)	67 (97.10)		
Diagnosis, *n* (%)				*χ*^2^ = 2.33	0.127
Imaging diagnosis	442 (91.13)	347 (90.13)	95 (95.00)		
Pathological diagnosis	43 (8.87)	38 (9.87)	5 (5.00)

**Table 4:** Comparison between different examination groups

	Total (*n* = 1165)	Chest low-dose plain scan (*n* = 375)	Abdominal normal-dose enhanced scan (*n* = 515)	Abdominal normal-dose plain scan (*n* = 275)	Statistic	*P*
Type, *n* (%)					*χ*^2^ = 4.20	0.122
Abnormal	485 (41.63)	140 (37.33)	225 (43.69)	120 (43.64)		
Normal	680 (58.37)	235 (62.67)	290 (56.31)	155 (56.36)		
Nodule characteristics, (%)					–	0.073
Malignant	7 (2.09)	0 (0.00)	6 (3.90)	1 (1.52)		
Benign	328 (97.91)	115 (100.00)	148 (96.10)	65 (98.48)		

### Quantitative Comparative Analysis of Internal and External Validation Outcomes

We conducted a comparison of the proposed AVQV within-model and cross-model. For the within-model comparison, we selected TotalSegmentator. TotalSegmentator, which is trained using the nnU-Net model, is capable of segmenting over 117 categories in CT images. It has been trained on a diverse range of CT images from various scanners, institutions, and protocols, making it highly applicable to a wide range of CT images. To comprehensively assess the performance of different architectures, the cross-model comparison included the V-Net model based on convolutional neural networks and the Swin-UNETR model based on the Transformer architecture. We reported all summary quantitative results in Tables 5 and 6. Our model achieved an average DSC of 0.87 ± 0.07 on the internal validation set and an average DSC of 0.88 ± 0.06 on the external validation set.

**Table 5:** Training and internal validation outcomes

**Category**	**Indicator**	**AVQV**	**TotalSegmentator**	**Swin-UNETR**	**V-Net**	**Statistic**	*P*
Total	DSC	**0.87 ± 0.07**	0.54 ± 0.26	0.86 ± 0.10	0.83 ± 0.11	*F* = 365.26	< 0.001
	HD95	4.02 ± 15.62	67.18 ± 145.29	**3.21 ± 7.77**	6.47 ± 18.71	*F* = 63.90	< 0.001
	RVE	**0.10 ± 0.11**	0.45 ± 0.28	0.11 ± 0.13	0.15 ± 0.15	*F* = 300.32	< 0.001
5 mm	DSC	**0.86 ± 0.06**	0.52 ± 0.27	0.84 ± 0.10	0.81 ± 0.11	*F* = 176.52	< 0.001
	HD95	**2.64 ± 5.75**	116.51 ± 191.37	3.61 ± 7.50	6.18 ± 12.48	*F* = 60.66	< 0.001
	RVE	**0.11 ± 0.11**	0.47 ± 0.31	0.13 ± 0.13	0.17 ± 0.16	*F* = 138.00	< 0.001
1 mm	DSC	**0.88 ± 0.08**	0.57 ± 0.24	0.87 ± 0.09	0.85 ± 0.10	*F* = 195.67	< 0.001
	HD95	5.40 ± 21.26	17.86 ± 28.68	**2.81 ± 8.03**	6.76 ± 23.37	*F* = 16.54	< 0.001
	RVE	**0.08 ± 0.11**	0.43 ± 0.25	0.10 ± 0.14	0.13 ± 0.15	*F* = 167.82	< 0.001
Normal	DSC	**0.86 ± 0.07**	0.51 ± 0.29	0.85 ± 0.09	0.83 ± 0.09	*F* = 226.55	< 0.001
	HD95	4.34 ± 18.88	88.93 ± 172.40	**2.33 ± 3.46**	5.35 ± 17.80	*F* = 47.48	< 0.001
	RVE	**0.10 ± 0.11**	0.48 ± 0.32	0.11 ± 0.12	0.15 ± 0.15	*F* = 171.41	< 0.001
Abnormal	DSC	**0.88 ± 0.08**	0.59 ± 0.21	0.86 ± 0.11	0.83 ± 0.13	*F* = 145.47	< 0.001
	HD95	**3.61 ± 9.92**	38.94 ± 92.86	4.36 ± 11.01	7.92 ± 19.80	*F* = 19.11	< 0.001
	RVE	**0.09 ± 0.12**	0.42 ± 0.23	0.12 ± 0.14	0.14 ± 0.16	*F* = 131.39	< 0.001
Chest low-dose plain scan	DSC	**0.86 ± 0.07**	0.38 ± 0.27	0.85 ± 0.08	0.83 ± 0.08	*F* = 301.36	< 0.001
	HD95	6.39 ± 23.74	144.24 ± 202.42	**2.76 ± 4.79**	5.61 ± 19.29	*F* = 59.29	< 0.001
	RVE	**0.11 ± 0.12**	0.59 ± 0.31	**0.11 ± 0.10**	0.13 ± 0.11	*F* = 222.93	< 0.001
Abdominal normal-dose plain scan	DSC	**0.86 ± 0.09**	0.57 ± 0.22	0.85 ± 0.10	0.83 ± 0.10	*F* = 78.24	< 0.001
	HD95	**2.31 ± 4.57**	27.25 ± 64.05	4.98 ± 13.04	7.85 ± 25.78	*F* = 7.87	< 0.001
	RVE	**0.11 ± 0.13**	0.43 ± 0.28	0.14 ± 0.18	0.16 ± 0.19	*F* = 40.19	< 0.001
Abdominal normal-dose enhanced scan	DSC	**0.89 ± 0.06**	0.67 ± 0.18	0.87 ± 0.11	0.83 ± 0.13	*F* = 88.51	< 0.001
	HD95	2.86 ± 8.89	21.66 ± 70.87	**2.70 ± 5.98**	6.51 ± 13.27	*F* = 9.13	< 0.001
	RVE	**0.08 ± 0.09**	0.35 ± 0.21	0.11 ± 0.12	0.15 ± 0.16	*F* = 95.12	< 0.001

The optimal results have been bolded in the table

*DSC* dice similarity coefficient, *AVQV* adrenal volume quantitative visualization tool, *1 mm* CT images with a slice thickness of 1 mm, *5 mm* CT images with a slice thickness of 5 mm

**Table 6:** External validation outcomes

Category	Indicator	AVQV	TotalSegmentator	Swin-UNETR	V-Net	Statistic	*P*
Total	DSC	**0.88 ± 0.06**	0.80 ± 0.19	0.87 ± 0.09	0.69 ± 0.14	*F* = 365.26	< 0.001
	HD95	**2.26 ± 4.51**	10.05 ± 28.69	4.86 ± 26.24	15.47 ± 49.32	*F* = 63.90	< 0.001
	RVE	**0.11 ± 0.11**	0.19 ± 0.23	0.11 ± 0.12	0.27 ± 0.18	*F* = 300.32	< 0.001
5 mm	DSC	**0.86 ± 0.06**	0.82 ± 0.10	0.85 ± 0.08	0.67 ± 0.14	*F* = 176.52	< 0.001
	HD95	**2.80 ± 5.01**	6.51 ± 22.91	5.13 ± 31.38	25.11 ± 68.11	*F* = 60.66	< 0.001
	RVE	**0.15 ± 0.11**	0.17 ± 0.14	0.15 ± 0.13	0.33 ± 0.18	*F* = 138.00	< 0.001
1 mm	DSC	**0.90 ± 0.06**	0.79 ± 0.26	0.89 ± 0.09	0.72 ± 0.14	*F* = 195.67	< 0.001
	HD95	**1.73 ± 3.88**	13.58 ± 33.16	4.60 ± 19.87	5.82 ± 6.99	*F* = 16.54	< 0.001
	RVE	**0.07 ± 0.08**	0.20 ± 0.29	0.08 ± 0.10	0.22 ± 0.18	*F* = 167.82	< 0.001
Normal	DSC	**0.88 ± 0.06**	0.81 ± 0.17	0.87 ± 0.07	0.69 ± 0.13	*F* = 226.55	< 0.001
	HD95	**2.05 ± 3.09**	7.64 ± 19.44	3.33 ± 10.65	14.68 ± 43.79	*F* = 47.48	< 0.001
	RVE	**0.11 ± 0.10**	0.17 ± 0.21	0.11 ± 0.11	0.26 ± 0.18	*F* = 171.41	< 0.001
Abnormal	DSC	**0.89 ± 0.06**	0.79 ± 0.24	0.87 ± 0.11	0.70 ± 0.15	*F* = 145.47	< 0.001
	HD95	**2.64 ± 6.30**	14.37 ± 40.02	7.62 ± 41.44	16.88 ± 58.06	*F* = 19.11	< 0.001
	RVE	**0.11 ± 0.12**	0.22 ± 0.27	0.12 ± 0.14	0.30 ± 0.20	*F* = 131.39	< 0.001
Chest low-dose plain scan	DSC	**0.87 ± 0.06**	0.57 ± 0.31	0.85 ± 0.07	0.63 ± 0.14	*F* = 301.36	< 0.001
	HD95	**3.53 ± 6.57**	30.48 ± 40.87	8.32 ± 23.12	16.26 ± 40.33	*F* = 59.29	< 0.001
	RVE	0.12 ± 0.11	0.47 ± 0.33	**0.11 ± 0.11**	0.27 ± 0.21	*F* = 222.93	< 0.001
Abdominal normal-dose plain scan	DSC	**0.87 ± 0.07**	0.85 ± 0.11	0.86 ± 0.12	0.67 ± 0.17	*F* = 78.24	< 0.001
	HD95	**2.07 ± 4.29**	6.86 ± 32.15	6.97 ± 43.16	22.02 ± 69.10	*F* = 7.87	< 0.001
	RVE	**0.10 ± 0.09**	0.11 ± 0.12	0.11 ± 0.13	0.28 ± 0.21	*F* = 40.19	< 0.001
Abdominal normal-dose enhanced scan	DSC	**0.89 ± 0.06**	0.87 ± 0.07	0.88 ± 0.06	0.73 ± 0.10	*F* = 88.51	< 0.001
	HD95	**1.88 ± 3.44**	3.95 ± 12.75	2.22 ± 5.29	11.17 ± 36.07	*F* = 9.13	< 0.001
	RVE	**0.11 ± 0.11**	0.12 ± 0.13	**0.11 ± 0.11**	0.27 ± 0.16	*F* = 95.12	< 0.001

The optimal results have been bolded in the table

*DSC* dice similarity coefficient, *AVQV* adrenal volume quantitative visualization tool, *1 mm* CT images with a slice thickness of 1 mm, *5 mm* CT images with a slice thickness of 5 mm

### Consistency Analysis of Volume Prediction with Manual Methods

The overall intraclass correlation coefficient (ICC) between AVQA and manual measurements was found to be 0.931 (95% CI 0.925, 0.936). Specifically, for chest low-dose plain scan, the ICC was 0.945 (95% CI 0.934, 0.953), for abdominal normal-dose plain scan, it was 0.889 (95% CI 0.873, 0.903), and for abdominal normal-dose enhanced scan, it was 0.93 (95% CI 0.922, 0.937). These excellent consistency values indicate that our tool can effectively replace manual measurements for volume determination.

### 3D Rendering

**Fig. 4:**
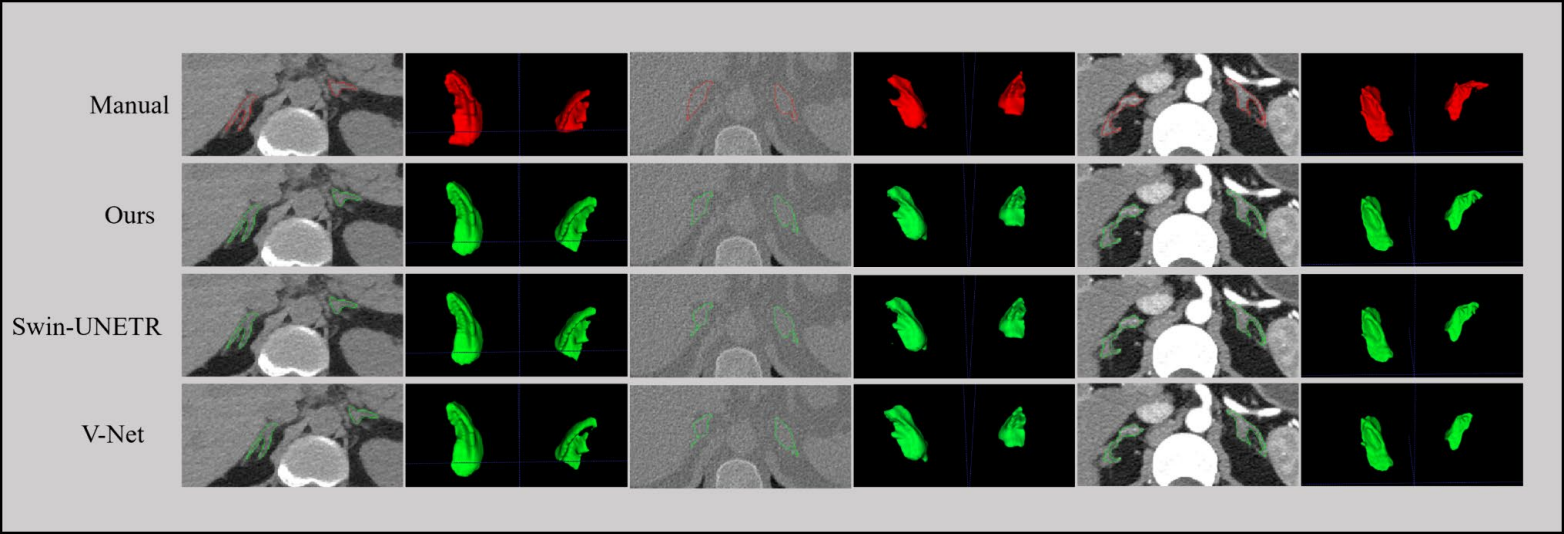
The red solid line represents manually delineated regions of interest ROI along the adrenal gland boundary. The green solid line represents the adrenal ROI generated using the model. The data for the three cases in the figure were obtained separately from different types of scans. Case 1 data was obtained from a chest low-dose plain scan, Case 2 data from an abdominal normal-dose plain scan, and Case 3 data from an abdominal normal-dose enhanced scan

We integrated two approaches utilizing ITK-SNAP software and the ITK-widgets visualization toolkit to display the 3D visualization outcomes of adrenal segmentation. ITK-SNAP is an open-source application designed for medical image segmentation and visualization; it has gained significant recognition in medical research, particularly in neuroradiology and radiation therapy planning. Its powerful 3D visualization capabilities allow for rendering segmented regions in three dimensions, supporting various color mappings and transparency adjustments, vividly depicting the contours of the adrenal glands. In contrast, the ITK-widgets package is an interactive toolkit for visualizing 2D/3D images within the Jupyter Notebook environment. This package not only facilitates the visualization of three-dimensional renditions of adrenal glands but also provides precise positional details within the abdominal context. The 3D output schematic is shown in Fig. 4.

## Discussion

In this study, we developed a highly automated versatile adrenal segmentation, volumetric quantification, and visualization tool that applies to complex clinical scenarios. In the internal validation set, the results demonstrate that the overall DSC of the model is 0.87, and for low-dose CT is 0.86. In the external validation set, the results indicate that the overall DSC of the model is 0.88, and for low-dose CT is 0.87. Compared to other segmentation models, including previous literature findings and the website model, our AVQV model demonstrates more accurate segmentation performance. The specific details can be found in Tables 5 and 6 and Appendix.

Developing a tool that can automatically segment adrenal glands in low-dose CT images and output their volumes is of great importance. Due to the crucial role of low-dose chest CT in screening for pulmonary nodules and reducing the risk of lung cancer, it has been widely applied in health check-ups for the general population [51, 52]. Subclinical or screening patients can benefit from accurate monitoring of adrenal gland status through follow-up examinations or check-ups, enabling early detection of changes. Additionally, it allows for acquiring a substantial amount of imaging information regarding adrenal morphology and volume from a large population of healthy individuals enabling the establishment of normative ranges for adrenal size based on big data, addressing the issue of the lack of standardized clinical criteria and improving the applicability of indicators, which is also the focus of our forthcoming work. However, previous models did not incorporate such data, leading to some accuracy concerns. Therefore, we trained a model that can quantify adrenal volume based on low-dose CT scans. The DSC of our tool, when compared with manual measurements of adrenal volume in chest low-dose plain scan was 0.87. The DSC for regular-dose scans was 0.87, and for contrast-enhanced scans, it was 0.89 Thus, low-dose CT scanning can yield favorable results. Our tool provides a user-friendly and easily applicable indicator for clinical practice and related research.

Furthermore, our focus includes training models based on multiple machine types, parameters, and morphologies, which ensures the high generalizability of our tool, allowing it to be applied in complex and diverse clinical scenarios with confidence. Previous studies suffered overfitting issues caused by limited sample inclusion and a single imaging parameter, leading to poor generalization and limited model applicability. In contrast, our model incorporates CT data from five mainstream CT machines, varying doses and scanning protocols, datasets comprising both normal and abnormal adrenal morphologies, and data reconstructed with different slice thicknesses. This comprehensive approach addresses the complexities and variabilities encountered in clinical scenarios, establishing a fully automated segmentation and quantification tool that provides a reliable volumetric reference index. The nnU-Net-based model not only exhibits superior segmentation capabilities compared to other deep learning or similar methods but also addresses tasks that previously needed a certain level of data and domain expertise, such as manual data preprocessing tasks and neural network architecture engineering for each developed solution [33]. Due to the ability of the nnU-Net method to automatically optimize crucial model tuning parameters based on unique input data constraints, it is easier to implement. We recommend its usage, as it requires less computational technical expertise than other related image segmentation tasks.

Currently, the radiological diagnosis of adrenal hyperplasia and small nodules has been challenging in clinical practice [53–55]. A small organ in the abdomen, the adrenal gland exhibits variable morphology and position and is prone to adhesion with surrounding organs, posing diagnostic challenges. Our visualization outputs, shown in Fig. 4, can aid in diagnosing adrenal morphological abnormalities that are difficult to identify in cross-sectional or coronal views. Furthermore, they can assist clinicians in preoperatively assessing adrenal surgery or resection.

However, our study also has some limitations. Currently, our tool does not include other imaging modalities, such as MRI, which results in a particular deficiency in the generalization of our model in multimodal segmentation. Additionally, our model lacks longitudinal model comparisons.

## Conclusion

Our research demonstrates that our deep learning-based automatic segmentation tool, based on a large sample, multiple parameters, multiple machine types, multiple radiation doses, multiple slice thicknesses, multiple scanning modes, multiple phases, and diverse adrenal morphological samples, exhibits high accuracy, robustness, and generalizability on CT scan images. The model can be directly applied to clinical practice workflows, providing accurate volumetric measurements and visualizations without manual preprocessing steps to meet the clinical requirements of adrenal disease diagnosis, screening, monitoring, and preoperative visualization assistance.

## Appendix

The following table shows the performance of the literature models:

**Table:**

Author (year)	**Core methodology/model used**	**Dataset size/type**	**Results**
Bhole et al. (2013) [25]	Conditional random field	22	AC = 86.59%
Chai et al. (2015) [24]	Multi-scale sparse representations	5/the tumor contour CT images	AC = 90.31% ± 2.97%
Koyuncu et al. (2018) [26]	Region growing techniques	32/the tumor arterial and portal phase dynamic CT images	DSC = 71.03% ~ 88.42%
Zhang and Li (2019) [27]	Level set methods	5	DSC = 82.18% ~ 99.62%(2D)
Robinson-Weiss et al. (2022) [28]	U-Net based segmentation model	274/venous phase abdominal CT	DSC = 80.0%
Kim et al. (2022) [29]	U-Net based segmentation model	308/contrast-enhanced abdominal CT	DSC = 69.9% ~ 70.6%
Luo et al. (2021) [30]	Two-level cascade deep neural network	348/venous phase of enhanced abdominal CT scans with primary aldosteronism before medication	DSC = 87.42% ± 5.88%
Li et al. (2024) [31]	Multi-level context-aware network	65/50	DSC = 71.34% ~ 75.29%
